# The Inflammasome in Host Defense

**DOI:** 10.3390/s100100097

**Published:** 2009-12-28

**Authors:** Gang Chen, Joao H.F. Pedra

**Affiliations:** Center for Disease Vector Research and Department of Entomology, University of California-Riverside, Riverside, 92521, CA, USA; E-Mail: gangc@ucr.edu

**Keywords:** inflammasome, nod-like receptors, innate immunity

## Abstract

Nod-like receptors have emerged as an important family of sensors in host defense. These receptors are expressed in macrophages, dendritic cells and monocytes and play an important role in microbial immunity. Some Nod-like receptors form the inflammasome, a protein complex that activates caspase-1 in response to several stimuli. Caspase-1 activation leads to processing and secretion of pro-inflammatory cytokines such as interleukin (IL)-1β and IL-18. Here, we discuss recent advances in the inflammasome field with an emphasis on host defense. We also compare differential requirements for inflammasome activation in dendritic cells, macrophages and monocytes.

## 1. Introduction

A quick immune response defines life and death for the host. To detect infection quickly, the immune system relies on innate immunity, the first line of defense against microbial infection that engages adaptive immunity [1]. Several families of innate immune receptors have been described [2,3]. These receptor families include Toll-like receptors (TLRs), retinoic acid-inducible gene I-like receptors (RLRs), and nucleotide-binding oligomerization domain-like receptors (NLRs). TLRs are membrane bound receptors located either in the plasma membrane or on vesicles of the endocytic pathway [4]. TLRs are widely studied and detect microbial pathogens that include viruses, bacteria, protozoa, and fungi. TLR recognition of pathogen-associated molecular patterns is done *via* extracellular leucine-rich repeat motifs that transmit signals through the cytoplasmic Toll-interleukin (IL)-1 receptor (TIR) domain [5]. RLRs are distinct from the TLR pathway and recognize viral RNA present within the cytoplasm [3]. RLRs proteins have a RNA-binding helicase domain and two amino (N)-terminal caspase recruitment domains (CARDs) for propagation to the interferon-regulatory factor and NF-κB signaling pathways [6,7].

**Figure 1.**
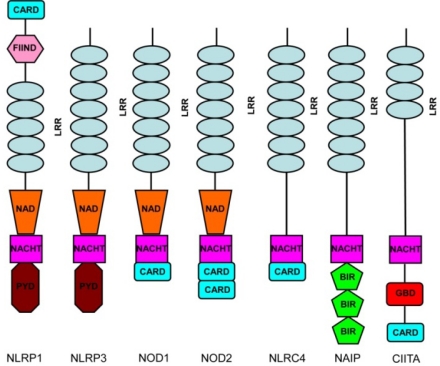
NLR domain structures. The domain structures of seven NLR members are shown. The CARD domain depicted in CIITA is only present in some splice forms. Abbreviations: BIR, baculovirus IAP (inhibitor of apoptosis protein) repeat; CARD, caspase-recruitment domain; CIITA, major histocompatibility complex (MHC) class II transactivator; FIIND, domain with a function to find; NLRC4/IPAF, interleukin 1β-converting enzyme protease-activating factor; LRR, leucine-rich repeat; NACHT, domain present in NAIP, CIITA, HET-E (incompatibility locus protein from *Podospora anserine*) and telomerase associated protein; NAD, NACHT-associated domain; NAIP, neuronal apoptosis inhibitor protein; PYD, pyrin domain; GBP, GTP binding domain.

NLRs are a family of intracellular sensors that have key roles in innate immunity and inflammation [8–11]. NLRs are defined by an architecture that contains a *N*-terminus, a middle nucleotide binding and oligomerization domain, and a leucine-rich repeat domain that is variable in the repeats composition and number (Figure 1). A number of NLR family members can form multiprotein complexes, called inflammasomes, and are capable of activating the cysteine protease caspase-1 in response to a wide range of stimuli including both microbial and self-molecules. NLRs induce the recruitment of the adaptor molecule ASC (apoptosis associated speck-like protein containing a CARD), leading to the processing and activation of pro-IL-1β and IL-18 through caspase-1. NLRs are mostly cytosolic and structurally related to the disease resistant protein family (R protein) [12]. In this review we will discuss the mechanisms by which the inflammasome is activated in cells. A greater emphasis will be placed on a broader function of the inflammasome in host defense. We will elaborate on the cross-talk between the inflammasome and other signaling pathways. We also review mechanisms of inflammasome activation according to the environment in which each cell type is located. We make a distinction between findings observed in macrophages, dendritic cells and monocytes. We apologize in advance to those whose work could not be cited due to space constraints.

## 2. The Inflammasome

**Figure 2.**
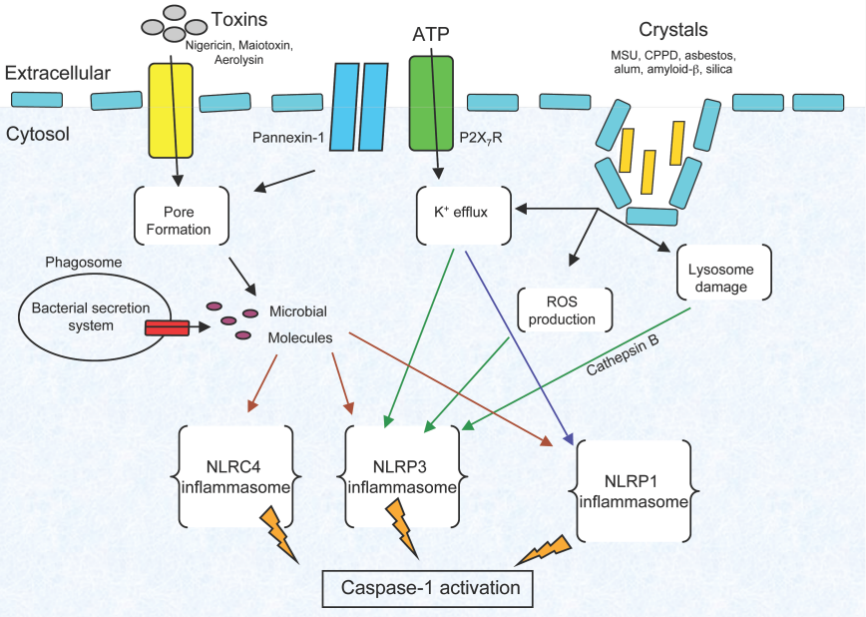
Mechanisms of inflammasome activation. Three different NLRs regulate the inflammasome. The NLRC4 inflammasome is triggered by microbial components secreted by bacterial secretion systems. The NLRP1/NALP1 inflammasome is associated with susceptibility to the *Bacillus anthracis* lethal toxin. Microbial toxins and danger signals trigger the NLRP3/NALP3 inflammasome. Crystals cause lysosome damage and protein leakage to the cytosol. Response to the NLRP3/NALP3 inflammasome triggers reactive oxygen species and potassium efflux although it is not clear how the signaling cascade operates.

In humans the NLR family is composed of 22 members [10]. They are divided into subfamilies according to their *N*-terminal effector domains. The effector domains lead to inflammatory caspase or NF-κB activation. In humans, 14 NLR members share a pyrin effector domain. Five other members are part of the NOD subfamily. CIITA, NLRC4/IPAF and the BIR-containing NAIP form the remaining NLR members. Earlier nomenclature was confusing; however, a standardized nomenclature has recently been proposed, and we will use it throughout this review [13]. NLR molecules have been shown to form a complex with caspase-1 and the adaptor molecule ASC termed the inflammasome. The term inflammasome was coined to describe a complex of proteins that activates caspase-1 and the cytokine IL-1β. Inflammasome is derived from the word inflammation and the suffix “some”, which is used to define molecular complexes [10]. This term was also chosen to highlight functional similarities with another platform that triggers apoptosis—the apoptosome [14]. The central effector molecule of the inflammasome is the cysteine protease caspase-1 that, upon activation cleaves cytosolic pro-IL-1β, pro-IL-18 to their active forms. Recent studies have shown that the NLRP1, NLRP3, and NLRC4 inflammasomes have a clear role in host defense (Figure 2). These distinct inflammasomes are described below.

### 2.1. The NLRP1/NALP1 Inflammasome

The NLRP1/NALP1 inflammasome (also known as DEFCAP, NAC, CARD7 and CLR17.1) is formed as a reconstituted complex enriched for caspase-1, caspase-5, and the adaptor molecule ASC [15]. Reed and colleagues have demonstrated that the minimal components for inflammasome activation are NLRP1/NALP1 and caspase-1 in the presence of ribonucleotides [16]. The bacterial cell wall component muramyl dipeptide (MDP) strongly activates the inflammasome *in-vitro*, although direct binding of MDP to NLRP1/NALP1 was not demonstrated.

The adaptor molecule ASC was not required for NLRP1/NALP1 inflammasome activation although its presence did enhance caspase-1 activation. NLRP1/NALP1 also plays a role in anthrax immunity [17–23]. *Bacillus anthracis* lethal toxin (LT) can induce caspase-1-dependent cell death of macrophages. Boyden and Dietrich revealed that the *NLRP1/NALP1b* gene, one of three paralogues of *NLRP1/NALP1* in mice, is responsible for macrophage susceptibility to LT [18]. Hsu *et al.* have also shown that NLRP1/NALP1 plays a role in LT-induced caspase-1-dependent IL-1β secretion in response to intact *B. anthracis* [24]. This study demonstrated that NOD2 was required for *B. anthracis*-induced IL-1β secretion, suggesting that NOD2 may interact with the NLRP1/NALP1 inflammasome. Interestingly, the group of Tschopp has demonstrated that mouse effector and memory CD4^+^ T cells abolish macrophage inflammasome-mediated caspase-1 activation and interleukin-1β. Inhibition of the inflammasome is observed for NLRP1/NALP1, NLRP3/NALP3 and the NLRC4/IPAF protein complexes [25].

### 2.2. The NLRC4/Ipaf Inflammasome

The NLRC4/Ipaf inflammasome (also known as CARD12, CLR2.1 and CLAN) is important for caspase-1 activation in response to *Salmonella*, *Pseudomonas*, *Legionella*, *Listeria, Anaplasma* and *Shigella* [26–38]. NLRC4/IPAF responds to the presence of flagellin within the macrophage cytosol during infection by *Salmonella* [27,30,39], *Legionella* [26,40–47] and *Pseudomonas* [29,33,34]. *Shigella,* however, does not express flagellin, suggesting that NLRC4/IPAF may signal through a molecule independently of flagellin expression [36,37]. For *Legionella* infection, Vance and colleagues [47] have generated mice deficient in the intracellular sensor Naip5/Birc1e/NLRB1 and have elegantly demonstrated that these mice fail to activate the inflammasome in response to a segment of the carboxyl terminus of flagellin. Signaling mechanisms that form the NLRC4/IPAF inflammasome has been recently expanded. Using *Legionella* as a model pathogen, Akhter *et al.* [48] have demonstrated that caspase-7 was activated downstream of the NLRC4/ IPAF inflammasome and required caspase-1 activation. Caspase-7 activation was mediated by flagellin and required a functional Naip5 [49,50].

### 2.3. The NLRP3/NALP3 Inflammasome

The NLRP3/NALP3 (also known as cryopyrin, CIAS1, PYPAF1 and CLR1.1) is the best-studied inflammasome and is activated by chemically and structurally diverse stimuli—*i.e.*, malarial hemozoin, crystal structures and fungal toxins, viral RNA, among others [8,51–68]. LPS and extracellular ATP stimulate caspase-1 in a NLRP3/NALP3 dependent fashion [54,55]. ATP activates the purinergic receptor P2X7, which recruits another hemichannel—pannexin-1. Pannexin-1 forms a larger pore of the P2X7 purinergic receptor upon activation and is important for caspase-1 activation [69–72]. Pannexin-1 channels can be activated by high extracellular K^+^. Activation of pannexin-1 channels by extracellular K^+^ occurs in cells voltage-clamped to the resting membrane potential. Therefore, it cannot occur due to the depolarization resulting from the elimination of the transmembrane K^+^ gradient. It is unclear whether K^+^ activation of pannexin 1 channels occurs *in vivo.* Despite a well-documented role of K^+^ ions in the NLRP3/NALP3 inflammasome *in-vitro*, the mechanism of action is poorly understood.

The NLRP3/NALP3 inflammasome assembly occurs when potassium efflux inducing agents such as nigericin, *Listeria* LLO, *Aeromonas* aerolysin, and maiotoxin induce the formation of plasma membrane pores [53,55]. *Mycobacterium tuberculosis*, through its ESX-1 secretion system, has been shown to activate the NLRP3/NALP3 inflammasome [73]. DNA, bacterial RNA and the antiviral imidazoquinoline compounds R837 and R848 also induce the NLRP3/NALP3 inflammasome [57,59]. However, four independent groups have recently demonstrated that immune detection of foreign dsDNA in the cytoplasm occurs primarily through the AIM2—a member of the HIN-200 family of IFN-inducible proteins that recruits ASC and caspase-1 [74–77]. Particulate matter such as gout and pseudogout-associated uric acid crystals, monosodium urate and calcium pyrophosphate dihydrate and fungal infection activate the NLRP3/NALP3 inflammasome in a P2X7-independent manner [49,56,78–82]. Silica and aluminum salt crystals activate the NLRP3/NALP3 inflammasome through phagocytosis [60]. Crystals induced lysosome swelling, damage and leakage of proteins to the cytosol. The inhibition of a lysosomal protein -cathepsin B- leads to a significant decrease in NLRP3/NALP3 activation [58,83,84]. Two events that appear to be required for both toxin- and crystal-mediated NLRP3/NALP3 inflammasome activation are efflux of intracellular potassium and the generation of reactive oxygen species (ROS). A decrease in intracellular potassium concentrations may be required for assembly of the NLRP3/NALP3 inflammasome, however, the precise role of potassium in this process is unknown [85]. Similarly blockade of ROS production using chemical inhibitors has been shown to mitigate the ability of silica, asbestos and ATP to activate the NLRP3/NALP3 inflammasome [85–88]. Ng and colleagues demonstrated that uric acid crystals could directly engage cholesterol rich cellular membranes resulting in the activation of a Syk kinase-dependent signaling cascade [89]. Syk kinase signaling was also coupled to the NLRP3/NALP3 inflammasome for anti-fungal host defense.

## 3. Inflammasome Activation Requirements

Dendritic cells, macrophages and circulating monocytes must respond to microbial threats and are essential to host defense. Inflammasome activation in monocytes and dendritic cells and macrophages are different. Two fundamental differences exist for inflammasome activation in dendritic cells, macrophages and circulating monocytes. While monocytes have constitutively activated caspase-1, the activation of caspase-1 in dendritic cells and macrophages needs to be induced.

**Figure 3.**
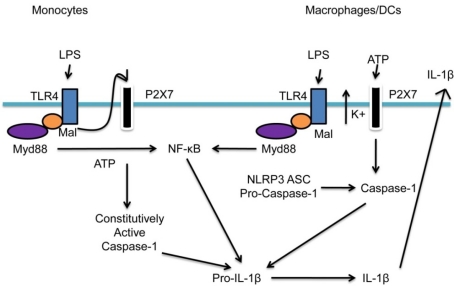
Differential requirements for inflammasome activation in immune cells. Caspase-1 is constitutively activated in circulating monocytes and release mature IL-1β after a single stimulation with TLR ligands such as LPS. IL-1β secretion is induced by endogenously released ATP. Dendritic cells and macrophages, however, need double stimulation: the first stimulus may be a TLR ligand (e.g., LPS), which induces transcription of IL-1β *via* NF-κB. The second stimulus (e.g., exogenous ATP, bacterial toxins) triggers NLR and caspase-1 activation followed by IL-1β secretion.

Furthermore, monocytes are capable of releasing endogenous ATP [90]—an important molecule for inflammasome activation, whereas the lack of endogenous ATP by macrophages and dendritic cells renders these cells incapable of IL-1β secretion with one single stimulus. Netea and colleagues [91] have recently clarified these mechanisms. Monocytes only need a single TLR stimulus for IL-1β secretion, whereas dendritic cells and macrophages need a double stimulation—TLR (e.g., LPS) and NLR (e.g., ATP) stimuli (Figure 3). The reason for this differential regulation is poorly understood. Current evidence suggests an adaptation of each cell type to their respective environment. Circulating monocytes function in a pathogen-free environment and must promptly respond to a microbial threat. Dendritic cells and macrophages are confined to a non-sterile environment and are constantly exposed to microbial pathogens. Therefore, a second checkpoint mechanism is necessary to avoid deleterious inflammation.

The difference in inflammasome activation requirements does not seem to be restricted to macrophages, dendritic cells and monocytes. In contrast to monocytes and macrophages, in which low intracellular K^+^ triggers inflammasome activation, neurons and astrocytes activate caspase-1 by high intracellular K^+^ [92]. In epithelial cells, which do not secrete high levels of IL-1β, caspase-1 enhances lipid metabolism and is required for optimal growth of the intracellular bacterium *Chlamydiae* [68]. Mast cells were identified as the main cell population responsible for IL-1β in the skin of cryopyrin-associated periodic syndrome patients. Unlike normal mast cells that required stimulation with pro-inflammatory stimuli for IL-1β production, resident mast cells from cryopyrin-associated periodic syndrome patients constitutively produced IL-1β *via* the NLRP3 inflammasome [93]. Collectively, these findings demonstrate that each cell type commits the inflammasome to the immediate environment in which they are located. This differential requirement for inflammasome activation operates to prevent accidental or uncontrolled inflammation, which may have devastated consequences for the host. A better understanding of the inflammasome activation in cells other than macrophages and dendritic cells may have significant ramifications for the treatment of infectious diseases and inflammatory disorders.

## 4. Conclusions

The cytoplasm was thought for a long time to be a safe haven for pathogenic microbes. Once viruses and intracellular bacteria evaded cell surface innate immune receptors, they could establish themselves into the cell interior, and replicate in it unabated. Recent findings, however, have challenged this line of thinking. Newly discovered cytoplasmic defenses such as NLRs have been show to preserve the cytosolic environment against pathogenic microbes. The field of NLRs and the inflammasome has blossomed into an important area of innate immunity and inflammation. We have attempted to describe these latest findings with an emphasis on host defense. Several fundamental questions remain unanswered in the NLR and the inflammasome field. The first and foremost fundamental issue is related to whether NLR recognition of pathogen-associated molecular patterns is direct or indirect. A second issue relates to the scope of the studies in field of the inflammasome. Most studies have been focused on macrophages and dendritic cells and three types of inflammasome—*NLRP1/NALP1, NLRP3/NALP3*, and *NLRC4/IPAF* inflammasome. We reason that it is important to investigate other types of inflammasome in cells other than macrophages and dendritic cells for the safer design of therapeutics. A third issue that needs to be explored is the connection of the inflammasome with adaptive immunity. Although a few papers have emerged recently [25,94], it is poorly established how the inflammasome engages adaptive immunity in host defense. There are many roles for the inflammasome yet to be examined. The importance of the inflammasome in host defense makes this protein complex an attractive target for therapeutic intervention in microbial immunity.

## Abbreviations

TLRs: Toll-like receptors
RLRs: Retinoic acid-inducible gene I-like receptors
NLRs: Nod-like receptors
IL: interleukin
CARD: Caspase recruitment domain
ASC: apoptosis associated speck-like protein containing a CARD
TIR: Toll-interleukin-1 receptor domain
BIR: baculovirus IAP (inhibitor of apoptosis protein) repeat
NLRC4/IPAF: interleukin 1β-converting enzyme protease-activating factor
LRR: leucine-rich repeat
NACHT: domain present in NAIP, CIITA, HET-E (*Podospora anserine* incompatibility locus protein) and telomerase associated protein
NAD: NACHT-associated domain; NAIP–neuronal apoptosis inhibitor protein
PYD: pyrin domain
GBP: GTP binding domain
NF-κB: nuclear factor kappa-light-chain-enhancer of activated B cells
ESX-1: (ESAT-6 secretion system-1)
ROS: Reactive oxygen species
LPS: lipopolysaccharide.
